# Association of supine and upright blood pressure differences with head-up tilt test outcomes in children with vasovagal syncope

**DOI:** 10.3389/fped.2025.1438400

**Published:** 2025-04-16

**Authors:** Mohammad Reza Khalilian, Mohammad Mehdi Nasehi, Fargol Farahmandi, Farzaneh Farahmandi, Tahmineh Tahouri, Parynaz Parhizgar

**Affiliations:** ^1^ Department of Pediatrics, Shahid Beheshti University of Medical Sciences, Tehran, Iran; ^2^ Pediatric Neurology Research Center, Research Institute for Children’s Health, Shahid Beheshti University of Medical Sciences, Tehran, Iran; ^3^ School of Medicine, Shahid Beheshti University of Medical Sciences, Tehran, Iran; ^4^ Shahid Modarres Educational Hospital, Shahid Beheshti University of Medical Sciences, Tehran, Iran

**Keywords:** neurally mediated syncope, pediatric syncope, vasovagal, head-up tilt test, syncope

## Abstract

**Introduction:**

Vasovagal syncope (VVS) is the most common childhood neurally mediated syncope. This study aims to define the different hemodynamic patterns in pediatric cases of vasovagal syncope and the difference between supine and upright blood pressure (orthostatic blood pressure), and other factors affecting them.

**Methods:**

Medical records of 88 children with normal laboratory and subclinical tests referred to Mofid Hospital for head-up tilt testing were retrospectively evaluated. Eighty-five children, 31 (36.5%) boys and 54 (63.5%) girls, aged 10.72 ± 3.52 years (median 10; range 4–17), with vasovagal syncope, were enrolled in the study. Age, sex, baseline heart rate, baseline blood pressure, orthostatic blood pressure, provoking factors, symptoms, and frequency were recorded. The test protocol included 10 min of supine rest followed by 30 min of upright positioning at a 70-degree angle. Subjects were divided into groups according to their differing hemodynamic patterns based on modified VASIS criteria.

**Results:**

Our data showed a strong association between the difference in supine and upright blood pressure and positive HUTT results (*p* = 0.001). In the positive HUTT group, age had a higher probability of vasodepressor pattern in younger patients (*p*-value = 0.014). Also, a significant relationship is seen with the boys (*p*-value = 0.014, 0.038), which means that the male gender increases the probability of a positive tilt test.

**Discussion:**

Our study indicates that orthostatic blood pressure can predict HUTT in VVS children. Also, our results showed there is a higher chance of having a positive response to HUTT for the younger boys. Further studies in this field are warranted.

## Introduction

Reflex syncope is characterized by a transient loss of consciousness due to a sudden drop in cerebral perfusion pressure, resulting from impaired blood pressure autoregulation, which may cause vasodilation and/or bradycardia. The most prevalent type of reflex syncope is vasovagal syncope. This pathophysiology of vasovagal syncope is suggested to be decreased vascular tone, decreased cardiac output, increased total peripheral vascular resistance (TPVR) and reduced systemic vascular resistance (1–3). Vasovagal syncope (VVS) impacts approximately one-third of individuals (4). Diagnosis of VVS is based on clinical history and diagnostic features, including predisposing factors, prodromal symptoms, physical exam, and postdrome recovery and symptoms in the absence of heart disease (5). Hemodynamic patterns of vasovagal syncope is classified using modified VASIS criteria (6). Patients are divided into four subgroups based on arterial pressure and heart rate recorded during the tilt test. Type 1 patients have a mixed hemodynamic pattern where a decrease in blood pressure accompanies bradycardia, but the heart rate is not less than 40 beats per minute. Asystole may be present but lasts only for less than 3 s. Type 2A patients have a cardioinhibitory pattern, where the heart rate falls below 40 beats per minute for more than 10 s. Arterial blood pressure decreases before the heart rate decreases. Type 2B patients have a cardioinhibitory and asystolic pattern, where the heart rate drops below 40 beats per minute for more than 10 s, and asystole may occur but lasts for less than 3 s. Type 3 patients have a vasodepressor pattern where the heart rate does not decrease by more than 10% of its initial rate, even at the most prominent moment of syncope. However, blood pressure is significantly reduced (6).

The head-up tilt test (HUTT) is performed when a neurocardiogenic cause of syncope is suspected and heart disease has been excluded, helping to confirm a diagnosis of vasovagal syncope (VVS) (7, 8). HUTT has been utilized in children since 1990 to diagnose and assess treatment effectiveness and is considered the gold standard for identifying various forms of vasovagal syncope (9).

In our research, we assess the variation in blood pressure between the supine and upright positions, referred to as orthostatic blood pressure, as part of our evaluations prior to the HUTT.

This research seeks to explore the various hemodynamic patterns associated with vasovagal syncope in children who have experienced one or more episodes of this condition and compare them to those with negative outcomes, while also considering the factors that influence these results.

## Materials and methods

In this study, data from children who had vasovagal syncope and were referred to Mofid Children Hospital for the HUTT, were collected from 2008 to 2022. The age, sex, duration of symptoms, frequency of syncope, severity, provoking factors, symptoms during syncope, family history, baseline heart rate, baseline blood pressure, and orthostatic blood pressure during HUTT were collected in these patients.

Data such as the subjects’ symptoms, signs, laboratory testing, electrocardiogram (ECG), echocardiography, electroencephalogram (EEG), neurological exam, psychiatric exam, physical exam of head, eyes, ears, nose, and throat (HEENT), chest, abdomen, and heart were collected. In patients who had a positive HUTT, the minute of having symptoms and their symptoms were also collected. The institutional ethics committee approved the study protocol (ethics code: IR.SBMU.MSP.REC.1401.230).

### Exclusion criteria

Data from patients diagnosed with a chronic disease that could have affected their HUTT results or were not compatible with vasovagal syncope were not collected. All cases with symptomatic arrhythmias or other chronic underlying conditions such as congenital heart disease, cardiac hypertrophy, asthma, anemia, epilepsy, diabetes mellitus, and psychiatric disorders, or drugs affecting the autonomic nervous or cardiovascular system were excluded from the study.

### Conducting HUTT protocol

The head-up tilt test (HUTT) was performed according to standard guidelines to diagnose vasovagal syncope. Following a 4 h fasting period, the test was conducted in a quiet, dimly lit, and temperature-controlled environment. The HUTT protocol spans a duration of 50 min. Patients were initially placed in a supine position for 10 min, followed by a 70-degree tilt for 30 min. This angle was chosen based on recent guidelines to optimize the detection of true positive results while minimizing false diagnoses (10). Based on the patients’ results during the tilt test, baseline heart rate, baseline blood pressure, orthostatic blood pressure, and heart rate blood pressure during HUTT, the patients were divided into groups according to their differing hemodynamic patterns If there is no change in the hemodynamic pattern of patients (nor the blood pressure nor the heart rate) the test is considered as negative HUTT. Otherwise the patients have positive HUTT and are divided through modified VASIS criteria (6), which categorize patients based on their hemodynamic responses during a tilt test. There are four main types of hemodynamic patterns:

Type 1: This group experiences a mixed pattern where blood pressure drops along with bradycardia, but the heart rate remains above 40 beats per minute. If asystole occurs, it lasts for less than 3 s.
Type 2A: These patients show a cardioinhibitory pattern, where the heart rate falls below 40 beats per minute for more than 10 s. In this case, blood pressure decreases before the heart rate does.
Type 2B: Similar to Type 2A, but encompasses both cardioinhibition and asystole, with the heart rate again dropping below 40 bpm for more than 10 s. Asystole may occur, but it lasts less than 3 s.
Type 3: Patients in this group have a vasodepressor pattern; despite experiencing a significant drop in blood pressure, their heart rate does not decrease by more than 10% from the initial level, even at the onset of syncope.

These classifications help in understanding the different hemodynamic responses associated with vasovagal syncope.

### Statistical analysis

Statistical analysis was conducted using SPSS 26. Numerical data were presented as mean ± standard deviation or median (range), and rates as percentages. Binary logistic regression assessed normally distributed data between groups, while linear regression estimated the relationship between one independent and one dependent variable. The chi-square test and multinomial logistic regression evaluated relationships between variables. A *p*-value of less than 0.05 is considered significant.

## Results

### Characteristics of patients

The medical records of 88 children who underwent head-up tilt testing at Mofid Children's Hospital were retrospectively evaluated. These children had normal laboratory and paraclinical test results. Three patients who experienced discomfort during the test and had the test stopped were excluded from the study. The remaining 85 children, consisting of 31 (36.5%) boys and 54 (63.5%) girls, with a mean age of 10.72 ± 3.52 years (median 10; range 4–17), were enrolled in the study. They were diagnosed with vasovagal syncope. Based on the modified VASIS criteria, the 85 patients could be classified into four different hemodynamic patterns of vasovagal syncope. Forty-six (54.1%) patients had negative HUTT results, while 39 (45.9%) had positive HUTT results. Within the positive HUTT group, 23 (59%) exhibited a vasodepressor (type 3) pattern, and 16 (41%) showed a mixed (type 1) pattern. Type 2A (cardioinhibitory without asystole) and type 2B (cardioinhibitory with asystole) patterns were not observed in our patients.

Baseline blood pressure measurements were recorded for all patients. The mean blood pressure is 107.65/72.89 mmHg in the negative HUTT group, 104.35/70 mmHg in the vasodepressor group, and 106.69/71.94 mmHg in the mixed group.

### Relationship between HUTT results and other factors

Table 1 shows that there was no significant association between the baseline blood pressure of the patients and the HUTT results (all *p*-values > 0.05). Likelihood ratio tests, based on multinomial regression analysis, confirmed that baseline systolic blood pressure (SBP) and diastolic blood pressure (DBP) does not significantly affect HUTT results (SBP: *χ*^2^ = 49.004, df = 44, *p* = 0.279; DBP: *χ*^2^ = 33.120, df = 28, *p* = 0.231).

**Table 1:** Summary of patient characteristics and clinical findings in relation to HUTT results.

ITEMS	Total patients	Negative HUTT	Positive HUTT		*P*-value^†^ (95% confidence interval)	
			Vasodepressor	Mixed	Vasodepressor	Mixed
Age (years)	10.72 ± 3.52	11.28 ± 3.78	9.65 ± 3.03	10.63 ± 3.22	0.014* (0.678–0.957)	0.177 (0.734–1.059)
Sex (male/male %)	85 (31/36.5%)	46 (12/36.1%)	23 (11/47.8%)	16 (5/50%)	0.014* (0.064–0.728)	0.038* (0.067–0.957)
DOS (month)	21.07 ± 29.09	22.5 (0.5–120)	12.57 (0.25–60)	28.93 ± 38.53	0.459 (0.906–1.046)	0.937 (0.937–1.073)
Onset of symptoms (minutes)	18.31 ± 6.8	–	19.7 ± 6.2	16.31 ± 7.3	0.130 (0.978–1.193)	0.130 (0.838–1.023)
Frequency (total known events)	2.91 ± 2.1	2.67 ± 2.1	3.0 ± 2.2	3.44 ± 2.3	0.544 (0.851–1.358)	0.231 (0.907–1.498)
SHR (BPM)	90.4 ± 15.09	89.59 ± 15.76	92.91 ± 11.9	89.13 ± 17.53	0.805 (0.687–1.621)	0.960 (0.636–1.538)
UHR (BPM)	94.21 ± 14.82	92.89 ± 15.73	97.3 ± 12.21	93.58 ± 15.74	0.965 (0.665–1.532)	0.861 (0.676–1.598)
DELTA HR	3.8 ± 3.61	3.30 ± 3.17	4.391 ± 2.55	4.43 ± 5.6	0.238 (0.943–1.264)	0.275 (0.930–1.292)
SSBP (mmHg)	106.58 ± 9.17	107.65 ± 9.9	104.35 ± 7.69	106.69 ± 8.86	0.406 (0.884–1.051)	0.669 (0.923–1.133)
USBP (mmHg)	99.89 ± 9.76	103.35 ± 9.5	94.83 ± 8.36	97.25 ± 8.5	0.056 (0.815–1.003)	0.437 (0.861–1.067)
SBP reduction	6.68 ± 3.07	4.30 ± 1.60	9.52 ± 1.78	9.43 ± 1.63	0.001* (2.454–12.770)	0.001* (2.359–12.496)
SDBP (mmHg)	72.44 ± 5.96	72.89 ± 6.69	71.43 ± 4.31	72.56 ± 5.94	0.898 (0.897–1.100)	0.434 (0.825–1.086)
UDBP (mmHg)	69.09 ± 6.13	71.02 ± 6.51	66.57 ± 4.63	67.19 ± 5.16	0.473 (0.789–1.116)	0.857 (0.835–1.161)
DBP reduction	3.34 ± 2.19	1.86 ± 1.25	4.86 ± 1.5	5.37 ± 2.02	0.922 (0.378–2.927)	0.548 (0.487–3.873)

Multinomial logistic regression indicates a significant reduction in systolic and diastolic blood pressure in the positive HUTT group (*p* = 0.001). Likelihood ratio tests, based on multinomial regression analysis, confirmed that baseline systolic blood pressure (SBP) and diastolic blood pressure (DBP) does not significantly affect HUTT results.

Multinomial logistic regression indicates that the reduction in systolic (SBP) and diastolic blood pressure (DBP) from standing to supine is significantly greater in the positive HUTT group (*p* = 0.001). The mean time to symptom onset in the positive group showed no significant correlation to HUTT result (all *p*-values > 0.05). The mean frequency of syncope episodes does not significantly affect HUTT results (*p* = 0.231). The average duration of symptoms prior to HUTT had no correlation to HUTT outcomes (all *p*-values > 0.05). The analysis revealed no significant correlation between baseline blood pressure and HUTT outcomes (all *p*-values > 0.05).

HUTT, head up tilt test; DOS, duration of symptoms; HR, heart rate; SHR, supine heart rate; UHR, upright heart rate; BPM, beats per minute; BP, blood pressure; SSBP, supine systolic blood pressure; USBP, upright systolic blood pressure; SDBP, supine diastolic blood pressure; UDBP, upright diastolic blood pressure.

Data are provided as mean ± std.deviation.

^†^ *p*-values are computed versus negative group.

* *p*-value < 0.05 shows significant relation.

**Figure 1:**
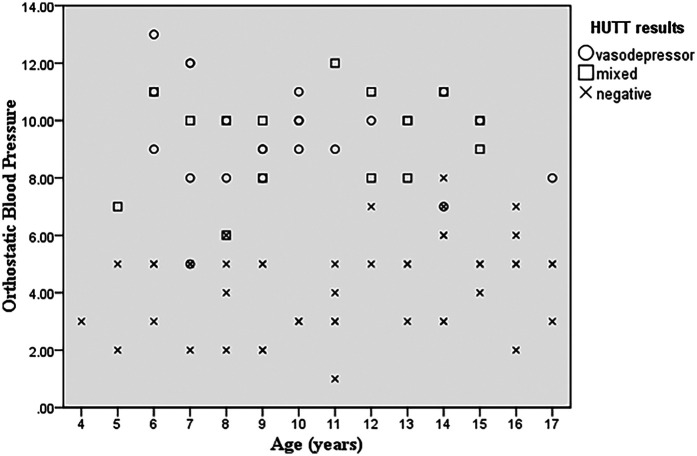
Scatterplot graph of orthostatic blood pressure and age of patients and HUTT results suggesting that with an increase in orthostatic blood pressure, the occurrence of a positive tilt test is higher.

Multinomial logistic regression analysis was used to examine the predictive value of changes in systolic blood pressure (SBP), diastolic blood pressure (DBP), and a combination of both (orthostatic blood pressure) from supine to standing. The reduction in SBP from standing to supine was significantly higher in the positive HUTT group. The *p*-value of 0.001 indicates a highly significant relationship, suggesting that with an increase in orthostatic blood pressure, the probability of a positive tilt test is 1.70 times higher (Figure 1). The results of the multinomial logistic regression analysis reveales a significant correlation between increased orthostatic blood pressure and positive tilt test outcomes. With a *p*-value of 0.001, the analysis showes a 1.72-fold increase in the likelihood of a vasodepressor pattern (OR = 5.598; 95% CI: 2.454–12.770) and a 1.69-fold increase for a mixed hemodynamic pattern (OR = 5.429; 95% CI: 2.359–12.496), indicating a strong association with elevated orthostatic blood pressure.

Forty-six (54.1%) of the patients had negative HUTT results, while 23 (59%) had a vasodepressor pattern and 16 (41%) had a mixed pattern. None of our patients exhibited a cardioinhibitory pattern. As shown in Table 2, younger age is associated with a higher probability of a vasodepressor pattern (*p*-value = 0.014, 95% CI:0.678–0.957). Both the vasodepressor (*p*-value = 0.014, 95%CI:0.064–0.728) and mixed groups (*p*-value = 0.038, 95%CI:0.067–0.926) showed a significant relationship with male gender indicating that male gender increases the likelihood of a positive tilt test. Boys have a higher incidence of a positive response to the head-up tilt test (HUTT) in our study (*p*-value = 0.038).

**Table 2:** Association of age and sex of participants with hemodynamic patterns in head-Up tilt test responses.

Positive HUTT		B	Std. Error	Wald	df	*P* value	Exp(B)	95% confidence interval for Exp(B)	
								Lower bound	Upper bound
Vasodepressor	Sex	−1.537	.622	6.098	1	.014	.215	.064	.728
	Age	−.216	.088	6.046	1	.014	.806	.678	.957
Mixed	Sex	−1.391	.670	4.305	1	.038	.249	.067	.926
	Age	−.126	.093	1.823	1	.177	.881	.734	1.059
a. the reference category is: negative.									

The data indicate that a younger age correlates with a higher probability of exhibiting a vasodepressor pattern (*p*-value = 0.014). Additionally, both the vasodepressor and mixed groups demonstrated a significant association with male gender (*p*-values = 0.014 and 0.038, respectively), suggesting that male gender increases the likelihood of a positive outcome on the tilt test. Notably, boys showed a higher incidence of positive responses to the Head-Up Tilt Test (HUTT) in this study (*p*-value = 0.038).

**Figure 2:**
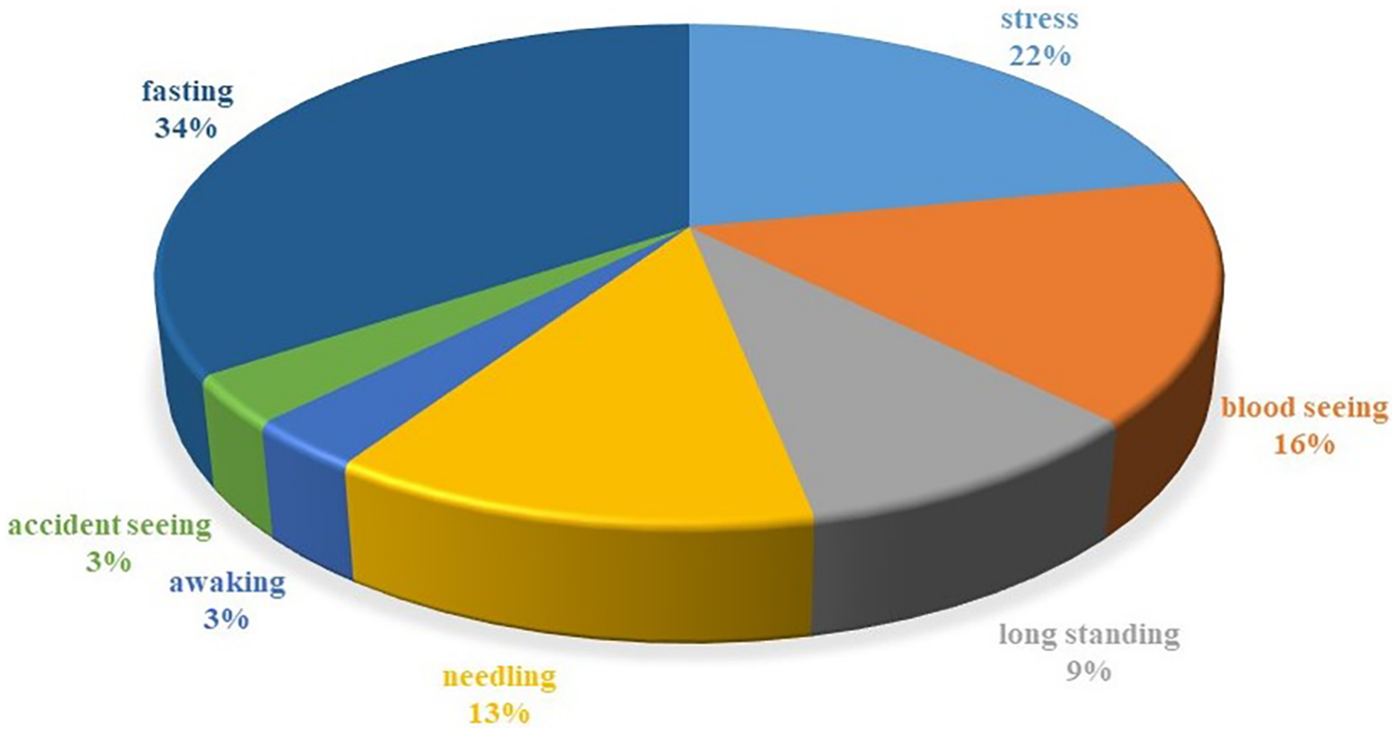
Piechart of the frequency of predisposing factors for syncope in patients. This graph illustrates the predisposing factors for syncope in patients, with fasting (34%) and stress (25%) being the most common. Among HUTT-positive participants, symptoms included pallor (69.3%), nausea (64.1%), dizziness (35.9%), and sweating (30.8%). Additionally, 32.9% experienced prodromal symptoms, primarily pallor (78.5%) and nausea (42.8%). This highlights the significant role of fasting and stress in syncope cases.

As shown in the pie chart of the frequency of predisposing factors for syncope in patients (Figure 2), the most common predisposing factors for syncope in our patients were fasting and stress (34% and 25%, respectively). Also the most common symptoms in the HUTT-positive group during the active phase of the trial were: pallor (69.3%), nausea (64.1%), dizziness (35.9%), and sweating (30.8%). In our research, we found that 32.9% of the patients reported experiencing prodromal symptoms. The most prevalent symptoms included pallor (78.5%), nausea (42.8%), headache (21.4%), sweating (14.2%), tonic posture (7.1%), and incontinence (3.5%), listed in order of frequency.

As shown in Table 1, the mean time to the onset of symptoms in the positive group was 18.31 ± 6.825 min, with a median of 18 (range 7–30) minutes. There is no significant correlation between the timing of symptom onset and disease severity in the patients (all *p*-values > 0.05). The mean frequency of syncope episodes in our patients was 2.91 ± 2.1, but this is not significantly associated with the HUTT results (all *p*-values > 0.05).

The average duration of symptoms experienced prior to the HUTT was 21.07 ± 29.09 months, spanning from under a month to as long as 5 years, with a median of 12 months. The findings indicate that the length of time patients were symptomatic does not correlate with the outcomes of the HUTT, as all *p*-values were above 0.05. Only 2% of individuals in our study had a positive family history of syncope. Due to this small proportion, we were unable to thoroughly evaluate the potential influence of familial syncope history on the outcomes of HUTT.

## Discussion

Although syncope may be attributed to cardiac diseases in older patients (11), the majority of syncope in young individuals is due to postural fainting or vasovagal syncope (VVS) (12). VVS is characterized by a vasovagal response, a significant shift of blood volume in the venous system, and the combination of vasodilation and vagal-induced bradycardia, resulting in hypotension and temporary loss of consciousness (2, 13).

A peak in vasovagal syncope incidence observes around the age of 15, with females having more than twice the incidence rate compared to males (14–19). The most common age for the first occurrence of vasovagal syncope is 13 years (20). In our study, the mean age of VVS cases was 10.72 ± 3.52 years (median 10; range 4–17). Our research indicates that male participants exhibited a greater frequency of positive responses to the head-up tilt test (HUTT) in this study (*p*-value = 0.038). Furthermore, the data suggest that a younger age correlates with an increased likelihood of vasodepressor-type syncope in individuals who tested positive on the HUTT (*p*-value = 0.014).

A study conducted by van Dijk et al. revealed that asystole occurred in 42% of the cases, with cardioinhibitory responses noted in 8.6%. Additionally, bradycardia observed in 43% of patients during the head-up tilt test (HUTT) (21). In a study by Kolarczyk et al., positive HUTT results were observed in children with VVS, with 45% having a mixed pattern, 51.7% having a vasodepressor pattern, and 3.3% having a cardioinhibitory pattern (17). In a study conducted by Yozgat et al. involving 400 children experiencing unexplained syncope, the hemodynamic response patterns associated with vasovagal syncope revealed intriguing results. Specifically, there were nine instances (2.2%) classified as type 3, ten instances (2.5%) as type 2A, two instances (0.5%) as type 2B, and a notable 17 instances (4.25%) categorized as type 1 (mixed). These findings highlight the diverse hemodynamic responses in pediatric patients, underscoring the complexity of diagnosing and understanding syncope in this population (22). As in this study 54.1% of the patients had negative HUTT results. In positive test HUTT resullts 59% of patients had a vasodepressor pattern and 41% had a mixed pattern. None of our patients exhibited a cardioinhibitory pattern.

A report suggests that patients with reflex syncope present with a hemodynamic profile that differs from the general population, displaying lower systolic blood pressure (SBP) and higher diastolic blood pressure (DBP) and heart rate (23). Yamanouchi et al. found that the vasovagal group experienced greater decreases in stroke index and ejection fraction during tilting compared to normal cases (13). Our study demonstrated a strong positive relationship between the difference in supine and standing blood pressure readings and a positive tilt test (*p*-value = 0.001). The orthostatic blood pressure mode in the positive HUTT group was double that of the negative HUTT group. Multinomial logistic regression revealed a significant association between elevated orthostatic blood pressure and positive tilt test outcomes, with a 1.72-fold increase in vasodepressor pattern and a 1.69-fold increase in mixed hemodynamic pattern.

Previous studies show that the difference between supine and upright heart rate in VVS cases is significantly greater than in the control group (24). Our data support an average increase of 3.8 ± 3.61 in heart rate among our VVS cases. There was no relationship observed between the change in heart rate and the pattern of a positive HUTT. This lack of correlation suggests that other factors may be influencing heart rate changes in VVS cases, and further research may be necessary to explore these dynamics more thoroughly.

Wieling et al. indicate that young individuals frequently experience prodromal signs and symptoms prior to the onset of spontaneous vasovagal syncope. Their research identified nausea, epigastric discomfort, and sweating as common prodromal symptoms associated with reflex syncope. The authors elucidated that these prodromal symptoms are linked to heightened autonomic control (16). The clinical presentation of vasovagal syncope can vary widely among young patients. The trigger for one event may be emotional, while another event could be postural. Furthermore, vasovagal episodes may occur without a identifiable trigger. Benign vasovagal episodes can also occur during daily activities such as playing, walking, cycling, and even during vigorous exercise (16). Among our patients, 28.2% had predisposing factors for syncope, and this rate increased to 43.5% among patients with a positive response to the tilt test. In Zhang et al.'s study, the positive response group exhibited the following predisposing factors before syncope: sudden awakening in the morning, prolonged standing, or emotional stress (19) and in Kolarczyk et al.'s study, the most common predisposing factors were change in body position and persistent standing and after effort in VVS children (17). In this research, the most common predisposing factors were fasting, stress, blood exposure, needle procedures, and prolonged standing, respectively.

The preliminary evaluation derived from patient history is crucial for diagnostic assessment, management, and risk stratification. According to the European Society of Cardiology (ESC) guidelines, vasovagal syncope (VVS) is highly probable if syncope is provoked by factors such as pain, fear, or standing, and is associated with characteristic symptoms like pallor, sweating, and/or nausea (4). In line with previous studies (25–27), our findings showes that 32.9% of patients experienced prodromal symptoms, with pallor, nausea, headache, and sweating being the most common.

The average duration of a positive tilt test in VVS patients in our study was 18.31 min. This duration represents the average time from the start of the tilt test to the onset of symptoms in patients. In Ghariq et al.'s study, only 2% of patients developed syncope after 3 min of the tilt test (18), whereas 2.3% of our cases exhibited symptoms during the initial 7 min of the tilt test. The mean time of symptom onset in the positive group was 18.31 ± 6.825 with a median of 18 (7–30) minutes. There is no relationship between the minute of symptom onset and disease severity in patients (all *P*-values > 0.05), and the more severe the disease, the less likely it is to have an effect on the time the patient becomes symptomatic during the tilt test.

Previous studies indicate that between 19% and 90% of patients with vasovagal syncope (VVS) have a positive family history of the condition. However, the incidence of VVS in these patients does not exceed that of the general population, which is estimated to be around 35%–39% (28–32). This discrepancy may be attributed to the variability in data from previous studies conducted in different populations and environments. Factors such as genetic diversity, lifestyle choices, and health care access can all influence the prevalence and reporting of a positive family history of vasovagal syncope (VVS). Additionally, differences in study methodologies, including sample size and diagnostic criteria, might contribute to the observed inconsistencies. VVS is considered to have a complex inheritance involving multiple genes and potential environmental factors, and it is most commonly inherited in an autosomal dominant manner (33). In 2020, a genome-wide association study (GWAS) that included diverse patient groups (those experiencing syncope and collapse compared to healthy participants) identified a specific polymorphic variant (rs12465214) linked to the condition at a genome-wide significant level in the 2q32.1 locus, that subsequently confirm with an independent sample (34). Among our cases, 2% had a positive family history of syncope which is not in the line with the previous studies. Among our cases, 2% had a positive family history of syncope, which does not align with previous studies, potentially indicating a limitation related to our sample size. We hypothesize that the lower percentage of familial cases in our study could be influenced by several factors, including the genetic background of our cohort and the environmental context in which these patients live. Furthermore, it may be worthwhile to explore whether different subtypes of VVS exhibit varying patterns of inheritance, as this could help clarify the complexities of its genetic underpinnings.

Finally, we would like to express our sincere gratitude to the children who participated in this project as well as their parents. Also the original data is available and can be provided upon request to interested researchers.

## Conclusion

In conclusion, our findings suggest that orthostatic systolic blood pressure (SBP) is a significant predictor of head-up tilt test (HUTT) outcomes in children with vasovagal syncope (VVS). Specifically, in our study, an increase in orthostatic blood pressure correlates with a 1.72-fold and 1.69-fold rise in the likelihood of experiencing vasodepressor and mixed hemodynamic responses, respectively. Furthermore, our findings suggest a higher probability of a positive response to HUTT among younger males. Nonetheless, due to the small sample size in this study, caution should be exercised in generalizing these findings to the wider population. Therefore, we strongly advocate for further research involving a larger and more diverse cohort to thoroughly explore the hypotheses presented in this study.

## Data Availability

The datasets presented in this article are not readily available because they are securely retained by the authors. Requests to access the datasets should be directed to the corresponding author.
